# Health needs of women victims of sexual violence in search for legal abortion ^*^

**DOI:** 10.1590/1518-8345.5834.3532

**Published:** 2022-04-29

**Authors:** Danyelle Leonette Araújo dos Santos, Rosa Maria Godoy Serpa da Fonseca

**Affiliations:** 1 Secretaria Estadual de Saúde Pública, Natal, RN, Brasil.; 2 Universidade de São Paulo, Escola de Enfermagem, São Paulo, SP, Brasil.

**Keywords:** Legal, Abortion, Induced, Abortion, Sexual Violence, Violence Against Women, Health Services Needs and Demand, Gender and Health, Aborto Legal, Aborto Induzido, Violência Sexual, Violência contra a Mulher, Necessidades e Demandas de Serviços de Saúde, Gênero e Saúde, Aborto Legal, Aborto Inducido, Violencia Sexual, Violencia contra la Mujer, Necesidades y Demandas de Servicios de Salud, Género y Salud

## Abstract

**Objective:**

to understand the health needs that emerge on the path followed toward legal abortion by women who have suffered sexual violence.

**Method:**

an exploratory, descriptive and qualitative study, whose theoretical framework was the conceptual field of Collective Health, based on the Marxian conception of needs. The study participants were ten women who requested legal abortion at a reference service located in São Paulo. A semi-structured interview script was used for data collection. The data were submitted to content analysis with the support of the WebQDA software. The analytical categories used were health needs and gender.

**Results:**

despite the fact that abortion was identified as a primary need, the data revealed other needs felt by women, related to the health-disease process and with an emphasis on mental health, work, financial situation, the exercise of motherhood, access to information, autonomy, sisterhood and welcoming in the health services.

**Conclusion:**

the properly human needs were in greater evidence in the study, surpassing merely biological needs. The results point to the importance of co-responsibility of the health services with women, aiming to minimize vulnerabilities and to effectively implement reproductive rights.

## Resumo

**Objetivo:**

compreender as necessidades em saúde que emergem durante a rota percorrida por mulheres que sofreram violência sexual para realização do aborto legal.

**Método:**

estudo exploratório, descritivo, qualitativo, cujo referencial teórico foi o campo conceitual da Saúde Coletiva, tendo como base a concepção marxiana de necessidades. Participaram do estudo dez mulheres que solicitaram aborto legal em um serviço de referência localizado em São Paulo. Para coleta de dados utilizou-se roteiro de entrevista semiestruturado. Os dados foram submetidos à análise de conteúdo com apoio do software WebQDA. As categorias analíticas utilizadas foram necessidades em saúde e gênero.

**Resultados:**

apesar de a efetivação do aborto ter sido apontada como necessidade primaz, os dados revelaram outras necessidades sentidas pelas mulheres, relacionadas ao processo saúde-doença com ênfase na saúde mental, ao trabalho, à situação financeira, ao exercício da maternidade, ao acesso à informação, à autonomia, à sororidade e ao acolhimento nos serviços de saúde.

**Conclusão:**

as necessidades propriamente humanas estiveram em maior evidência no estudo, superando necessidades meramente biológicas. Os resultados apontam para a importância de corresponsabilização dos serviços de saúde junto às mulheres, visando minimizar vulnerabilidades e efetivação de direitos reprodutivos.

## Resumen

**Objetivo:**

comprender las necesidades de salud que surgen en el camino que recorren las mujeres que han sufrido violencia sexual para realizarse un aborto legal.

**Método:**

estudio exploratorio, descriptivo, cualitativo, cuyo marco teórico fue el campo conceptual de la Salud Colectiva, basado en la concepción marxista de las necesidades. Participaron del estudio diez mujeres que solicitaron el aborto legal en un servicio de referencia ubicado en San Pablo. Para la recolección de datos se utilizó una guía para la entrevista semiestructurada. Los datos fueron sometidos a análisis de contenido con la ayuda del software WebQDA. Las categorías analíticas utilizadas fueron necesidades de salud y género.

**Resultados:**

a pesar de que el aborto fue identificado como la principal necesidad, los datos revelaron que las mujeres tienen otras necesidades que se relacionan con el proceso salud-enfermedad principalmente la salud mental, el trabajo, la situación económica, el ejercicio de la maternidad, el acceso a la información, la autonomía, la sororidad y la acogida en los servicios de salud.

**Conclusión:**

las necesidades humanas son las que más se destacaron en el estudio, superando a las necesidades meramente biológicas. Los resultados señalan que es importante que exista corresponsabilidad entre los servicios de salud y las mujeres, para reducir la vulnerabilidad y lograr la efectivización de los derechos reproductivos.

**Highlights**

(1) The search for legal abortion brought about properly human needs in women.

(2) Co-responsibility of the health services in the effective implementation of reproductive rights.

(3) Assistance based on horizontal communication is necessary, free from judgments.

## Introduction

The controversy surrounding the right to abortion is linked to ethical, moral, religious, social, gender and legal aspects. Since 1940, the Brazilian Penal Code, in its article 128, allows for abortions when there is a risk of death for the pregnant woman or when the pregnancy is the result of a rape^1^. In 2012, the Brazilian justice also decriminalized termination of anencephalic fetus pregnancies^2^.

Despite these legal exceptions, there are numerous obstacles faced by women to access the services that perform abortions in the country. In the case of pregnancies resulting from rapes, these obstacles become even greater due to the combination of stigmas attributed to sexual violence, women and abortion. It is known that the stigma of abortion is socially constructed and promotes judgments against women who choose to undergo such procedure, even in countries where legal restrictions do not exist, a fact directly related to the transgression of the roles assigned to women, whit motherhood as one of the pillars^3^ ^-^ ^4^. 

The stigma of abortion causes numerous barriers for women to access the services that perform legal abortion, perpetuating the silencing of accurate information about this practice^5^. The following stand out among these barriers: availability and quality of specialized services, accessibility, lack of knowledge about legality of the procedure and places for its conduction and emotional and cultural issues, as well as the health professionals’ attitude in the face of this demand^5^ ^-^ ^7^.

A Brazilian study that investigated the structure and functioning of hospitals responsible for the care of women who suffered sexual violence evidenced that, among the 68 institutions listed by the Ministry of Health, only 37 performed legal abortions and, among them, four never did so. The study also found a shortage of medical professionals available to perform the procedure, in addition to the requirement of a police report, judicial authorization and report from the Legal Medical Institute by some institutions, revealing excessive bureaucratization to access the right to abortion^8^. 

Faced with this scenario of difficulties and bureaucratization to the right to effectively perform an abortion, it is assumed that the obstacles experienced by the women who have suffered sexual violence in the search for legal abortion raise numerous health needs. The term “health needs” is not only understood as a user’s demand for the health service, but as an expression of socially and historically determined problems, arising from a collective structure^9^.

Health needs can be classified into visible and invisible, the former are related to the biological body and revealed through complaints, while the latter are linked to aspects that distance themselves from biological issues and demand care beyond the health services. Invisibilized needs require a keen eye on the part of health professionals to identify them, as their identification and care are beyond pre-established clinical scripts^10^.

It is recognized that violence triggers particular needs, most of the times, made invisible, which are directly related to the historical construction of the feminine in society and to the vulnerabilities to which women are exposed throughout their lives. In the case of women who have suffered sexual violence, it is assumed that the needs raised are not only linked to the violence suffered and to the pregnancy resulting from it, but also to the responses offered by the services that comprise the network to combat violence against women.

Thus, the current study started with the following research question: Which health needs are triggered during the path followed by women who have suffered sexual violence from the moment they decide to undergo a legal abortion until it is carried out? To answer this question, the study aimed at understanding the health needs that emerge during the path followed by women who have suffered sexual violence toward legal abortion.

By answering the research question, it is expected to obtain relevant elements that may contribute to the implementation and realization of public policies in the services that comprise the Network for the Assistance to Women in Situations of Violence, especially those available for performing legal abortions

## Method

### Type of study

An exploratory, descriptive and qualitative study, which adopted the conceptual field of Collective Health as a theoretical framework, based on the Marxian conception of needs^11^. In this way, historicity and dynamism were considered when analyzing individual and collective processes, determined by the economic, social, political and cultural aspects of society, allowing to understand reality and its contradictions in the structural, particular and singular dimensions, expanding the vision of a given phenomenon^12^.

### Study locus

The study was carried out in a reference hospital in the care of women who have suffered sexual violence and also legal abortions, located in the city of São Paulo, SP, Brazil. 

### Period

The data were collected over a period of three months, between July and September 2018. 

### Study population

The study population consisted of women who were pregnant as a result of sexual violence and who requested legal abortions. 

### Selection criteria

The inclusion criteria were women aged 18 years old or over, coming from municipalities located outside the Metropolitan Region of São Paulo or from other Brazilian states. It was defined that women with a previous diagnosis of severe psychiatric problems or with some cognitive impairment would be excluded from the research; however, it is noteworthy that during the data collection period there were no cases of women in these conditions.

The decision to interview women living outside the Metropolitan Region of São Paulo was based on the idea that residents of this region would face fewer difficulties accessing a service where they could request and carry out an abortion provided for by law, as the organization of the coping network of the women in situations of violence is better structured and given the constant disclosure in the São Paulo media about actions developed in the research development institution. 

### Participants

Ten women participated in the study, intentionally selected, and the sample was closed due to data saturation. 

### Data collection instruments

For data collection, an interview script was used, consisting of sociodemographic questions, aiming to characterize the participants, as well as guiding questions that served as a guide to understand the objective reality from the interviewees’ speeches.

### Data collection

Data collection took place using the in-depth interview technique. The invitation for the women to participate in the research took place after consultation with the social worker, at the time of requesting a legal abortion. It is noteworthy that no woman invited to participate in the research refused the invitation. 

All participants were interviewed at two moments: at the time of requesting the abortion and after performing the procedure or after a decision against the procedure. They took place in a private room of the service, being recorded on a digital voice recorder and lasting between 30 and 65 minutes. After the interview, all participants were asked about their desire to hear their speech, aiming to evaluate it and, if they wished so, they could change or exclude excerpts. No woman wanted to use this feature. 

It should be added that the audios of the interviews were stored in a password-protected folder on a personal computer of one of the researchers, who was solely responsible for handling them. In addition, in order to guarantee anonymity, the narratives of the interviewees were identified by the letter E for “*Entrevistada*” in Portuguese, followed by a number from 1 to 10, randomly assigned. 

During the entire data collection period, the constant presence of the researcher in the reference service allowed rich records in the field diary that contributed to a better understanding of the women’s experience during the process of requesting an abortion and subsequent hospitalization to carry out the procedure.

### Data treatment and analysis

Data analysis took place continuously, starting from data collection. Thus, at each interview, relevant themes were highlighted to elucidate the phenomenon, and those that presented similarity were grouped. Thus, in the absence of new elements in the treated material, theoretical data saturation was considered.

For data analysis, the procedures proposed by Bardin were followed^13^, aiming at the emergence of empirical categories, which were discussed in the light of the “health needs” and “gender” analytical categories. To support data analysis, the WebQDA software was used because it allows coding the qualitative research data in a structured and interconnected way, ensuring better organization and interpretation of the information in a faster and more systematic way^14^. 

### Ethical aspects

The study complied with all the ethical precepts proposed by Resolution No. 466, dated December 12^th^, 2012, of the National Health Council, obtaining approval from the Research Ethics Committee of the Nursing School of the University of São Paulo, under opinion No. 2,660,395, and by the Ethics Committee of the Reference Center for Women’s Health in the State of São Paulo, under opinion No. 2,661,916. It is noted that all participants signed the Free and Informed Consent Form after clarification of the objectives, benefits and risks of the research.

## Results

Among the study participants, five were between 20 and 29 years old and the others were between 30 and 39 years old. Most declared themselves white-skinned, with complete or ongoing higher education and with some religious belief, although not professing any specific religion. They worked in the formal and informal labor markets, with a family income varying between two and five minimum wages^*^. As for the cities of origin, all came from the southeastern region of the country, six living in the inland of the State of São Paulo and the others from Espírito Santo, Minas Gerais and Rio de Janeiro.

Regarding obstetric data, five participants had at least one child, while the others were experiencing their first pregnancy. Most had a gestational age ranging between nine and 13 weeks when they requested the legal abortion. Only two interviewees were in the second trimester of their pregnancies, with gestational ages of 17 and 23 weeks, respectively.

Analysis of the speeches revealed pregnancy as an extension of the sexual violence suffered, leading the participants to primarily translate their needs into the demand for abortion. Although they were not directly asked about the needs felt during the search for the abortion, emerging themes were identified in the reports that allowed for the emergence of four empirical categories.

In the first category, *needs related to the health-disease process of women in the face of unwanted pregnancies,* it was found that the news of the pregnancy triggered distress, reflecting negatively on the interviewees’ health care. The anguish regarding the outcome of the pregnancy was manifested in the form of anxiety, translated into needs aimed at preserving life, especially sleep and food. *[...] Basically, I stayed three days in bed [...] As I was very anxious, I had no appetite at all. I couldn’t sleep, I couldn’t eat, when I ate, I ate too much and felt sick.* (E2)*. I’m smoking twice as many cigarettes as I used to. I used to smoke two packs a day. I don’t even sleep with an urge to smoke. [...] I stay up all night, hovering between sleep and wakefulness [...]. I’m suffering.* (E5).

Some interviewees translated their needs exclusively into the field of mental health, emphasizing the importance of professional psychological support to deal with the experience of violence and abortion. *At night I have some crises that make me cry [...]. I can only feel extremely depressed, sad, lost in life [...]. I know I need help.* (E3)*. This is a very difficult decision to take. I don’t know which consequences it’ll have in my life [...]. I think I’ll have a lot of moral consequences, a very appealing and religious part.* (E4).

The empirical category called *needs related to the social production and reproduction processes* revealed how the interviewees’ routine was affected when they resorted to health services located in other cities to undergo the legal abortion. Social production, materialized in paid work, presented itself as an obstacle, given the women’s difficulty to be absent from work activities for a longer period of time than usual, a reality that points to the fragility and precariousness of the work relationships in a capitalistic context. *[...] the boss wanted to meet me to talk and have a meeting this week and I said that I couldn’t, that I’d take care of my health and that I couldn’t tell the reason why [...] he said he couldn’t have a person on the team who had a health problem [...].* (E3)*. This whole week I’m not going to work. [...] I’m worried here, afraid of losing my job [...] because I don’t work with a formal contract, I’m afraid of being dismissed.* (E5).

The financial difficulty also represented a significant obstacle for these women due to the expenses related to commuting and staying in another city. Whether due to the absence of health services or the scarcity of professionals who performed abortions in their cities of origin, the speeches revealed lack of responsibility from the services and health professionals, leaving to the women the sole responsibility of facing the problem. *I had to borrow some money. I owe my soul to come here for help.* (E3)*. It’s very complicated. If there was a hospital there, if I had support there in my city, I wouldn’t have had to come here, spend money I didn’t have. I had to borrow it in order to come. It’s horrible! It shouldn’t be like that!* (E8).

In relation to social reproduction, the participants who stated having children mentioned that the daily care of their offspring was an additional concern in the process of seeking an abortion. Thus, they reported feelings of guilt for understanding that they were transgressing the maternal role. *I left my kids there in my city with a neighbor of mine [...] I never left them like this, it’s the first time.* (E7)*. I even feel guilty because I have to take care of my son too. We’re doing an investigation to see if he has autism [...] I need to dedicate myself to him and, in this situation, I can’t do it.*(E10).

The third category, *needs related to women’s autonomy and citizenship,* showed that, despite the obstacles, the experience of sexual violence and unwanted pregnancy outweighed any obstacles encountered on the path to abortion. In this context, family support was found to be a need capable of strengthening the interviewees to contradict hegemonic social norms and adopt an active stance to seek abortion. In the meantime, attention is drawn to the appreciation given by some women to the support received by men from their family context. There were also those who chose not to seek family support due to religious dogmas, for example. *If it wasn’t for my cousin, what would I do? She knows other things, has a different mentality. Her fiancé has a different mentality. [...] Her fiancé kept telling me no one should judge me for anything. This coming from a man, wow, I felt more comfortable than my cousin supporting me.* (E2)*. My family is very conservative, all based on spiritualism [...]. I can’t tell them [...], they’re totally against abortion, even in case of rape. I didn’t know what to do.* (E3).

Access to information was also highlighted as a need that referred to autonomy. In general, those women surveyed were unaware of the legal possibility of performing an abortion when the pregnancy was the consequence of a rape, revealing this right only when they searched for clandestine abortions on the Internet. Some participants reflected on the difficulties that the social structure imposes for the effective implementation of this right in the reproductive field. *It was only by researching illegal methods on the Internet that I found out that this [abortion] is a woman’s right.* (E10)*. I know that by law every hospital has to do it, but that’s not how it goes. [...] Because Brazil is a country dominated by religion. So the very doctors who should perform [abortions] refuse to do so. [...] It’s a difficult situation.* (E8).

The experience of legal abortion also brought to light needs that referred to empathy and sisterhood. In general, after the abortion was performed, the interviewees relativized the potentials for wear out experienced by having received family support and, with that, strengthened their self-esteem, revealing a desire to encourage other women to enjoy this right in the future. *I really wanted to participate in a cause like this [...], if at some point I feel comfortable also to talk about what happened. [...] I live in a city that is not so small and this issue [abortion] is not addressed.* (E2)*. I’m very lucky. [...] Without support, I can’t imagine how difficult it must be. When I get over all this, I want to help other girls who go through these things.* (E4).

In the category called *needs related to welcoming in health services* precarious assistance and re-victimization of women by the professionals who assisted them were detected, a fact verified in the speeches, when they reveal the exposure, the embarrassment and the slowness to meet the demand for abortion. It is noted that those surveyed who resorted to more than one service along the way were more exposed to institutional violence. *I was tired of explaining the whole situation. Every time I went it was a new doctor and I had to explain everything all over again. They didn’t have a chart. I went through a different doctor each time. Always by the same procedures, but with different people.* (E4)*. They were passing me around like a ball, playing, playing, until someone was able solve it. That’s what I felt.* (E10).

For the women who lived in smaller municipalities, located in the inland of the states, there was fear of breach of professional secrecy and, thus, that sexual violence and pregnancy could become public knowledge. Fear of gender sanctions and community judgment made them not seek the health services. *In my city, because it’s inland, I don’t know, especially when it comes to rape, people judge you a lot. People are very prejudiced. And if I told the hospital, I might have it spread and also get a bad name.* (E3)*. I didn’t want anyone to know [...]. That’s why I didn’t go to the health center to do a pregnancy test. Because if I went, my mother would know.* (E6).

There were reports of inquisitive postures adopted by the health professionals, triggering precarious welcoming and minimizing any possibility of affective communication that would allow for qualified listening. *When I asked how the pregnant victims of abuse were treated, the social worker started asking me questions [...], she kept judging me [...]. I believe that those who work in the health area can’t think like that.* (E8).

Arrival of the interviewees at a hospital where they were finally treated enabled most of them to be provided with comprehensive and humanized assistance, based on qualified listening and resoluteness. However, in some speeches, it was highlighted that approval of the abortion request raised new needs that referred to the scarcity of information related to the procedure itself. In these cases, the approach based on technical language made communication ineffective, creating new gaps in welcoming. 

Thus, access to the information emerged as a constant need linked to women’s empowerment by reasserting their autonomy as subjects of rights. *I was afraid. I’ve never underwent any surgery. I didn’t know how the procedure was, so I imagined a lot of scalpels.* (E4)*. I don’t know how it works. So I’m worried that the procedure won’t work out.* (E9).

## Discussion

The analysis of the results revealed that the search for legal abortion is a path full of obstacles capable of triggering numerous health needs revealed as with potential for wear out in the women’s health-disease process. These hidden needs and, sometimes, minimized due to the urgency to carry out termination of pregnancy, were expressed in several vulnerabilities on the part of those surveyed.

The health needs felt and expressed by the participants were mainly linked to properly human needs, revealing overcoming of the needs for the conservation of life, whose focus is merely biological^11^. Although biological complaints were mentioned, it is believed that the women tried to translate more complex needs triggered by the context permeated by subjectivities. 

The results pointed to recognizing the need for specialized psychological assistance for the women to deal with the experience of violence and abortion. This data reproduces the idea disseminated within the scope of health services about violence being a phenomenon limited to mental health, as it escapes from the medicalizing focus, reinforcing the mind-body dichotomy^15^. Thus, the conception that such phenomena require the action of specialized professionals as a priority can create obstacles to care integrality and to the network articulation of services.

The nonexistence of services aimed at caring for women who have suffered sexual violence in the cities where the interviewees come from emerged as a needs-triggering element. The fact that most of these types of service are predominantly located in the capital cities of Brazilian states, not present in seven federative units^8^, forces women to leave their cities in search of the effective implementation of their rights. 

Geographic barriers to accessing abortion services have been evidenced in several countries and related to the low availability of services and professionals, especially in countries with restrictive laws, forcing women to travel long distances to terminate a pregnancy. These trips represent a burden, making the abortion experience more painful due to the direct and indirect costs of transportation, accommodation, food, distancing from work and/or studies and adjustments to ensure child care^16^ ^-^ ^19^.

The results revealed that the logistic burdens triggered needs related to social production, linked to fear of job loss and financial difficulties. It is known that paid work empowers women in overcoming gender inequalities through financial independence, having a close relationship with women’s autonomy and freedom in decision-making, thus turning to properly human needs^11^ ^,^ ^15^. 

Regarding the needs related to social reproduction, linked to motherhood, a gendered view of child care was verified. Although women have conquered a space in the public sphere, there is fear that the expansion of roles will lead to failures in the private context, especially in motherhood. The fear of not being able to fully perform motherhood triggered a feeling of guilt in the interviewees for fear that their choices would negatively influence their children’s well-being. This reality reinforces the social structure around the roles played by women in society and reveals the contradictions when they need to be divided between caring for their children and their personal desires^20^.

The obstacles and emotional conflicts experienced by the participants showed the strong influence of the social structure on the health needs. Not by chance were the needs that referred to autonomy and the exercise of citizenship translated into family support and access to information, revealed in the speeches as fundamental for encouraging decision-making for abortion. It is noted that some interviewees were surprised to have received support from men in their family context, attributing importance to male legitimation for their choice. Considering the structural dimension of society, it is understood that the value attributed to male support reinforces the androcentric culture and symbolizes a kind of social authorization of female choices in a patriarchal society. 

Regarding the difficulty acquiring information about legal abortion, some interviewees pointed out feelings that meant sisterhood when they expressed a desire to use their experiences in the search for abortion to encourage the autonomy of other women who undergo the same situation. The term sisterhood has been widely publicized in digital media and has contributed to the strengthening of feminist struggles. It is understood as an individual and collective experience that seeks to combat the patriarchal culture responsible for fighting conflicts between women, aiming at the establishment of supportive relationships that lead to female empowerment^21^.

Needs related to welcoming based on qualified listening, professional secrecy, support and resolute assistance were also mentioned by the participants. The speeches expressed frustration regarding the absence of a care network for victims of violence in the different municipalities of origin, lack of organizational structure of the services, and a judgmental attitude by the health professionals. This rupture of expectations reflected the difficulties of the health services in adequately responding to this type of demand, revealing the gender bias in the negative responses obtained in the different services and pointing out the resistance of these spaces in approaching the violence and abortion phenomena.

In the context of assistance to women in situations of violence, the health professionals’ attitudes greatly influence the assistance offered. Thus, welcoming should not be summarized as a simple conversation but presuppose comprehensive care, mediated by generalized knowledge capable of responding to these women’s needs. To this end, it is fundamental to use instruments that allow for a specific approach, with qualification of listening and translation of non-verbalized needs in the services^15^ ^,^ ^22^. 

In the current study, it was evidenced that the participants were attributed a position of passivity within the scope of the user-professional relationship, thus generating precarious welcoming, as they were deprived of relevant information that could significantly minimize needs that emerged along this path. It is therefore recognized that there are obstacles for female protagonism to be fully experienced in the sphere of sexual and reproductive health, especially when it comes to abortion. 

Also regarding welcoming, secrecy was pointed out as a need felt by the participants. Due to stigma, a large percentage of the women who undergo an abortion choose to keep it a secret for fear of being judged by others or having their desire to terminate the contested pregnancy^16^ ^,^ ^18^.

Although confidentiality is a users’ right and a duty of health professionals, the results showed that women from smaller cities feared having their privacy exposed. The fact that their social networks intertwine with those of the professionals reinforced the idea that there is no guarantee of anonymity when it comes to morally controversial social phenomena, such as abortion. In this context, it is necessary to reflect on how this barrier can directly influence women’s access to legal abortion and on how many give up on seeking this right, resorting to clandestine and unsafe procedures, risking lives. 

The combination of interpersonal and logistic barriers in the search for legal abortion reveals the urgent need to rethink the care network for women in situations of violence. A number of authors highlight as fundamental in this process the organization of services that meet this demand in more distant places, acting in an articulated way with other social devices, integrated by a multidisciplinary team capable of offering safe and confidential assistance^17^.

The limitation of this study lies in the fact that the interviews were carried out in the health service where the abortion was requested and performed, which may have somehow influenced the data on access and welcoming. Despite this, the results obtained contribute to expanding the knowledge in the field of Public Health about the phenomenon of abortion, as they correlate it with gender violence and discuss its interface with health needs. This integration allows for a new look at the phenomenon in question, contributing to the implementation of public policies aimed at fighting violence against women.

## Conclusion

The obstacles experienced by the surveyed women seeking legal abortions generated health needs not always expressed within the scope of the health services. In general, the women revealed properly human needs and, despite emphasizing the singular dimension, they had certain understanding of the influence of the social structure on the obstacles experienced by them along the path to abortion. 

The results showed that it is fundamental that the health services and professionals be co-responsible with women in the search for the effective implementation of their reproductive rights. Therefore, it is necessary that the meetings established within the scope of the health services are based on horizontal communication, free from the health professionals’ judgment and moral authority, with women being the real protagonists of their reproductive choices.

## Footnotes

* Current minimum wage = R$ 998.00, Brazil, 2019.
